# Transvaginal approach for resection of a low rectal leiomyoma: A case report

**DOI:** 10.1016/j.ijscr.2025.111984

**Published:** 2025-09-25

**Authors:** Tu Trong Doan, Duong The Pham, Cuong Van Nguyen, Thanh Tuan Tran, Hai Van Nguyen

**Affiliations:** a Department of Abdominal Surgery 2, Vietnam National cancer Hospital, Viet Nam; b Department of Radiation Oncology 2, Vietnam National cancer Hospital, Viet Nam

**Keywords:** Case report, Rectal leiomyoma, Transvaginal resection

## Abstract

**Introduction:**

Rectal leiomyomas are rare benign lesions with an estimated incidence of 1 in 2000 rectal tumors. The treatment of choice for these tumors is complete resection. Transvaginal approach is an appropriate method for tumors located in the anterior wall of the rectum. We herein report a case of a 31-year-old female patient treated by transvaginal resection for a lower rectal leiomyoma and also mention a literature review regarding this topic.

**Case report:**

A 31-year-old female patient was admitted to our hospital due to abdominal pain. She was diagnosed with a lower rectal leiomyoma and underwent transvaginal tumor resection. Postoperative course was stable and the patient was discharged after 5 days with no complications of vaginal fistula or sphincter dysfunction.

**Discussion:**

Leiomyoma is a common benign tumor that can be found in many locations in the body, but rarely occurs in the rectum. Large leiomyomas of lower rectum would normally require transanal, intersphincteric, or transperineal resection. However, these approaches had risks related to sphincteric dysfunction and anastomosis leakage. Transvaginal resection is a minimally invasive approach that is an appropriate method for tumors located in the anterior wall of the rectum.

**Conclusion:**

Transvaginal resection is a minimally invasive approach that may be considered for lower rectal submucosal tumors.

## Highlights

• Rectal leiomyoma is a rare benign tumor of rectum.
• Standard treatment is complete resection.
• Transvaginal resection may be considered for submucosal tumors located at the anterior wall of lower rectum.

## 1 Introduction

Leiomyoma is a benign tumor that can theoretically develop wherever smooth muscle is present, but rarely occurs in the rectum. Only about 200 cases of rectal leiomyoma have been reported worldwide, with an estimated incidence of 1 in 2000 rectal tumors [1]. Rectal leiomyomas often present with non-specific symptoms or are asymptomatic and are usually detected incidentally through endoscopy or diagnostic imaging methods. Immunohistochemical features of leiomyoma included positivity for desmin and alpha-smooth muscle actin and negativity for C-kit, CD34, and S100. Surgery is considered the best method of treatment for this disease. There are many approaches to remove the tumor: transanal, transperineal, transabdominal, transvaginal resection… Transvaginal resection is a minimally invasive approach that is an appropriate method for tumors located in the anterior wall of the rectum. We herein report a case of rectal leiomyoma that underwent transvaginal resection and review the literature about this topic. This work has been reported in line with the SCARE criteria [2].

## 2 Case presentation

A 31-year-old female with no medical history was referred to our hospital due to chronic pain in the lower abdomen. At the time, all vital signs were normal. Digital rectal examination detected a mobile mass with smooth surface and clear boundary located at the anterior wall of lower rectum within 3 cm from the anal verge. The patient denied any symptoms of bowel obstruction or hematochezia. The abdomen is soft, not distended, and no palpable mass was appreciated.

Pelvic magnetic resonance imaging revealed a 60x61mm in size tumor with clear boundary growing from the submucosa of the anterior wall of lower rectum with heterogeneous signal on T1W and T2W **(**Figs. 2**a,b)**. Routine lab data were unremarkable. CEA level was normal (CEA =3.6).

**Figs. 1:**
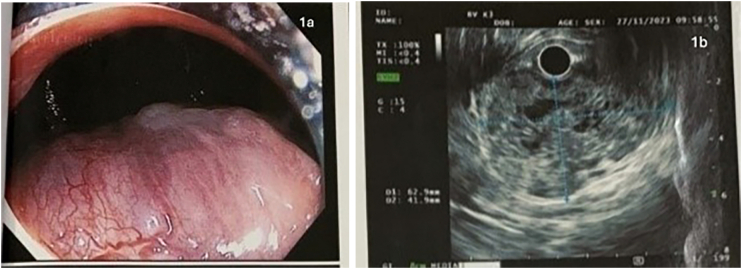
a,b: Images of rectal submucosal tumor by proctoscopy (1a) and endoscopic ultrasound (1b).

On the proctoscopy, there was a mucosa protruding mass with a smooth surface, about 4 cm in size **(**Fig. 1**a).** An endoscopic ultrasound showed a mixed echoic mass located in the anterior wall of the rectum, 63 mm × 42 mm in size, with no infiltration of the mesorectum **(**Fig. 1**b)**. A core needle biopsy via endoscopic ultrasound was performed. Microscopic evaluation of the tumor revealed spindle tumor cells. Immunohistochemistry results were positive for SMA, Ki-67 < 5 %, negative for CD34, S-100, CD117 and DOG-1 that confirmed leiomyoma.

**Figs. 2:**
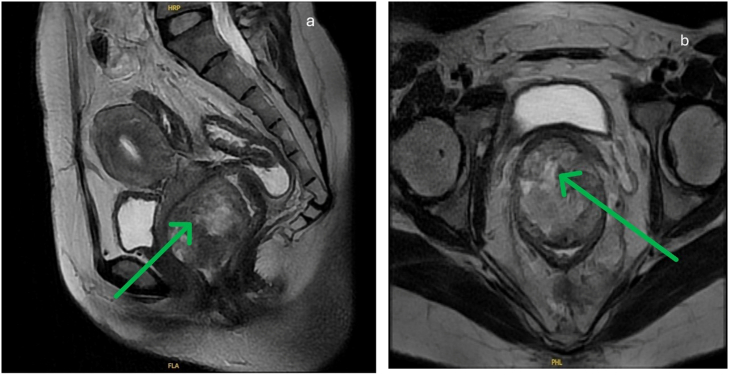
a,b: Pelvic magnetic resonance image showed a 63 mm × 42 mm tumor in the anterior wall of lower rectum (green arrow).

**Figs. 3:**
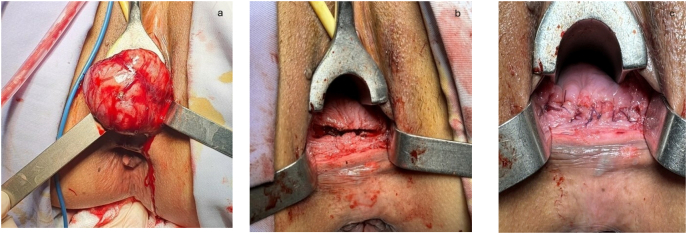
a,b,c: Removing the tumor through a horizontal incision in the posterior wall of vagina.

**Fig. 4:**
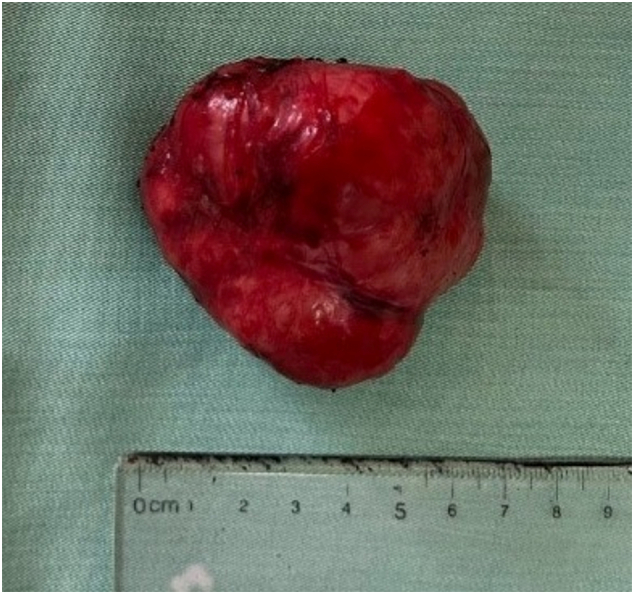
Post-operative image of specimen.

**Fig. 5:**
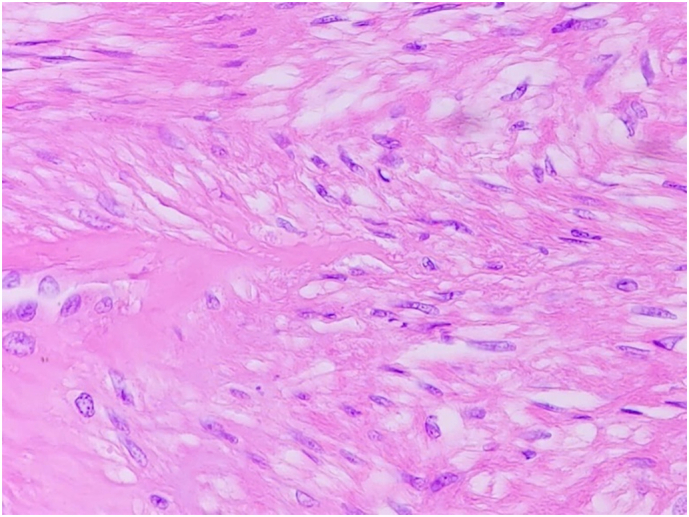
HE staining on microscopic evaluation revealed spindle tumor cells.

**Figs. 6:**
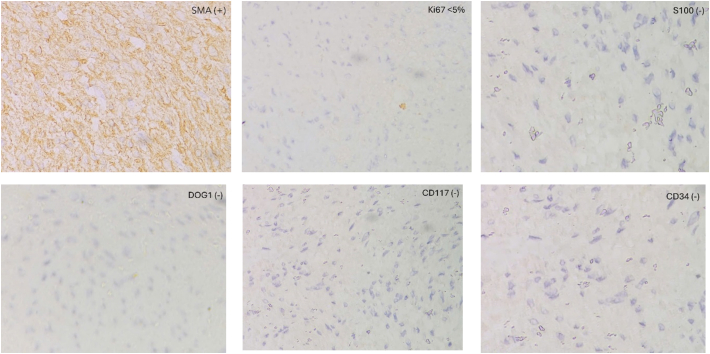
Immunohistochemistry results with SMA (+), Ki67 < 5 %, S100 (−), DOG1 (−), CD117 (−), CD34 (−).

The patient was diagnosed with lower rectal leiomyoma and was indicated for surgical resection. She was subjected to general anesthesia and placed in the lithotomy position. We made a 5 cm horizontal incision in the posterior vaginal wall to expose the rectovaginal septum. We exposed and mobilized the tumor from the surrounding tissue, and ensured that the capsule surrounding the tumor was kept intact during dissection **(**Fig. 3**a)**. Clearly defining the tumor boundaries and carefully dissecting the tumor capsule from the layers of the rectum is important for surgical removal of the entire tumor without rupture and preserving the integrity of the rectal mucosa. After removing the tumor, an important step is a digital rectal exam to check that the rectal mucosa was not damaged. The vaginal wall was reconstructed by continuous suture **(**Figs. 3**b,** 3**c).**

The total duration of surgery was 1 h with a minimal amount of blood loss (about 20 ml). Final pathology and IHC one more time confirmed the diagnosis of rectal leiomyoma (Fig. 4, Fig. 5, Figs. 6**).** The postoperative course was uneventful and the patient was discharged 5 days after surgery. One month later, the patient was followed up via a planned visit, she did not experience anal dysfunction or discomfort after surgery.

## 3 Discussion

Leiomyomas are benign tumors that can localize at any location in the body wherever smooth muscle is present. In the gastrointestinal tract, leiomyoma prefers to be in the esophagus, stomach and small intestine, and uncommonly appears in the rectum [3]. Rectal leiomyomas originate from the smooth muscle fibers of muscularis mucosa or muscularis propria of the rectal wall. They account for only 3 % of all types of GI leiomyomas, and approximately 0.1 % of rectal tumors [4].

Hatch analyzed 432 cases of leiomyomas in the rectum and anus from 1881 to 1996. Overall, rectal leiomyomas are most often discovered at the ages of 40 to 60 and are more common in males than females [5]. The clinical appearance of tumors can vary depending on size, location and tumor progression. Some patients present with changes in bowel habits, rectal bleeding, anorectal pain, or a palpable mass. But majority of rectal leiomyomas are usually asymptomatic and are detected incidentally through routine health check-ups.

Colonoscopy may help detect abnormalities in the colorectum. This procedure provides excellent visualization of the luminal aspect of mucosal lesions, but it has limitations in evaluating submucosal tumors. Transrectal endoscopic ultrasound (EUS) is the most accurate procedure for detecting and diagnosing submucosa rectal tumors, due to its high sensitivity and specificity. It is usually indicated as the next step following their detection by colonoscopy. EUS can provide information concerning origin, size, borders, homogeneity, and foci with echogenic or anechoic features. In addition, EUS can help to determine potential candidates for endoscopic resection. MRI allows the assessment of perirectal tissues and pelvic organs in addition to the entire thickness of the rectum, so it is effective for the evaluation of rectal submucosal tumors. The specific MRI characteristics of rectal leiomyomas have not been well described, it is reported that they are isointense to mildly hyperintense on T2-weighted images when compared to that of muscle [6,7].

In HE staining, leiomyomas are characterized by patterns of spindle cells, with eosinophilic or occasional fibrillar cytoplasm and distinct cell membranes. It is important to differentiate between leiomyomas and leiomyosarcomas, which are malignancy lesions characterized by large tumor cells, few stromal fibers, nuclear pleomorphism and increased mitotic activity. Leiomyomas in the lower part of the rectum are also usually misdiagnosed with GISTs in the MRI findings, endoscopy and endoscopic ultrasound. Immunohistochemistry analysis helps to differentiate leiomyoma and GIST. Leiomyomas are generally positive with SMA and negative with CD34 and CD117 [8].

Treatment for rectal leiomyoma may involve endoscopic removal or surgical resection, depending on tumor size and depth of invasion that can be identified by EUS or MRI. If the tumor is small and originates from the muscularis mucosae, endoscopic resection is an appropriate indication [9]. Surgical resection is the treatment of choice for tumors found deep within the submucosa. There are many approaches to remove the tumor: transanal, transperineal, transabdominal and transvaginal resection… The method of choice is decided depending on the characteristics of the tumor and the surgeon's experience. Transvaginal approach is a minimally invasive option that can avoid damaging the rectal mucosa and anal sphincter. It also offers a good visual field due to the large volume and the flexibility of the vagina. This approach is well-suited for GISTs or benign tumors on the anterior rectal wall with clear boundaries. In this particular clinical case, our patient was preoperatively diagnosed with a large leiomyoma confirmed by immunohistochemistry. The tumor located on the anterior rectal wall without invasion of rectal mucosa and other structures around the rectum. Therefore, transvaginal resection is the most appropriate method for this case. The patient underwent tumor enucleation without impairment of the rectal sphincter or rupture of the rectal mucosa, and the vaginal wall was repaired smoothly.

Vorobyov described 36 patients with rectal leiomyomas who underwent tumor resection from 1972 to 1990. Endoscopic resection was performed in twelve patients with tumors measuring below 1 cm. Tumors with a diameter of 2.5 to 5 cm were removed transanally in ten patients. One patient with tumor located in the anterior wall of rectum was resected transvaginally. Six patients underwent transperineal resection, whereas seven patients with large tumors underwent abdominoperineal extirpation or abdominoanal resection. Early postoperative complications were observed in 11 patients. Wound infection was the most common complication. The female patient who underwent tumor resection through vagina developed a rectovaginal fistula, which disappeared after conservative treatment [10]. In our case postoperative recovery was uneventful, the patient was discharged 5 days after surgery without fistula or sphincteric dysfunction.

## 4 Conclusion

Transvaginal resection is a minimally invasive approach that may be considered for lower rectal submucosal tumors.

## Consent

Written informed consent was obtained from the patient for publication of this case report and accompanying images. A copy of the written consent is available for review by the Editor-in-Chief of this journal on request.

## Ethical approval

The patient is anonymized and does not include any identifiable personal information. The manuscript was approved by ethical committee of Viet Nam National cancer hospital.

## Funding

The authors received no financial support for the research, authorship, and/or publication of this report.

## Author contribution

Tu Trong Doan: the main doctor conceived the original idea and operated the patient, revised manuscript.

Duong The Pham: operated the patient, followed up, wrote manuscript.

Cuong Van Nguyen: operated the patient, followed up, revised manuscript.

Thanh Tuan Tran: followed up, revised manuscript.

Hai Van Nguyen: followed up, revised manuscript.

## Guarantor

Tu Trong Doan

## Research registration number

Not applicable.

## Conflict of interest statement

The authors declare that they have no competing interests relevant to the content of this article.
